# The distribution and diversity of eukaryotic phytoplankton in the Icelandic marine environment

**DOI:** 10.1038/s41598-023-35537-2

**Published:** 2023-05-25

**Authors:** Mia Cerfonteyn, René Groben, Daniel Vaulot, Kristinn Guðmundsson, Pauline Vannier, María Dolores Pérez-Hernández, Viggó Þór Marteinsson

**Affiliations:** 1 Matís, Vinlandsleið 12, 113 Reykjavík, Iceland; 2 Faculty of Food Science and Nutrition, University of Iceland, Læknagarður, Vatnsmyrarvegur 16, 101 Reykjavík, Iceland; 3 Marine and Freshwater Research Institute, Fornubúðir 5, 220 Hafnarfjörður, Iceland; 4 Sorbonne Université, CNRS, UMR7144, Station Biologique de Roscoff, 29680 Roscoff, France; 5 Unidad Océano y Clima, Instituto de Oceanografía y Cambio Global, IOCAG, Universidad de Las Palmas de Gran Canaria, ULPGC, Unidad Asociada ULPGC-CSIC, Las Palmas de Gran Canaria, Spain

**Keywords:** Marine biology, Microbial ecology

## Abstract

Phytoplankton play a crucial role in the marine food web and are sensitive indicators of environmental change. Iceland is at the center of a contrasting hydrography, with cold Arctic water coming in from the north and warmer Atlantic water from the south, making this geographical location very sensitive to climate change. We used DNA metabarcoding to determine the biogeography of phytoplankton in this area of accelerating change. Seawater samples were collected in spring (2012–2018), summer (2017) and winter (2018) together with corresponding physico-chemical metadata around Iceland. Amplicon sequencing of the V4 region of the 18S rRNA gene indicates that eukaryotic phytoplankton community composition is different between the northern and southern water masses, with some genera completely absent from Polar Water masses. *Emiliania* was more dominant in the Atlantic-influenced waters and in summer, and *Phaeocystis* was more dominant in the colder, northern waters and in winter. The Chlorophyta picophytoplankton genus, *Micromonas*, was similarly dominant to the dominant diatom genus, *Chaetoceros*. This study presents an extensive dataset which can be linked with other 18s rRNA datasets for further investigation into the diversity and biogeography of marine protists in the North Atlantic.

## Introduction

Phytoplankton are the dominant primary producers in the ocean, and changes in their diversity and abundance have bottom-up consequences on higher trophic levels such as zooplankton and fish. Establishing the drivers behind these changes is critical for understanding the potential impacts of climate change on the marine food web. Drivers behind the biogeography of marine microorganisms are ocean currents, mesoscale dynamical features like eddies or upwelling filaments and local environmental conditions^1,2^.

Recent changes in oceanographic conditions have led to drastic shifts in phytoplankton dynamics, and recently we have already seen a poleward expansion of temperate phytoplankton into the European Arctic Corridor^3,4^. This range expansion of temperate phytoplankton, particularly the coccolithophore *Emiliania huxleyi*, has been linked with the north-eastward propagation of the Atlantic Water layer over the European Arctic Corridor (‘atlantification’^5,6^). The initial assumption was that this change in biogeography was due to increased water temperature, but Oziel et al.^7^ showed that it is mainly the acceleration of North Atlantic currents that is driving the *E. huxleyi* expansion. This shows that local environmental conditions are not always the main driver for changes in phytoplankton diversity, but also the manner in which phytoplankton groups are dispersed from their source environments.

In the North Atlantic and Arctic Oceans, assemblages of marine bacteria are clearly different over relatively short distances due to the meeting of different water masses^8,9^. Iceland is located in such an environment: cold polar and Arctic water from the north (via the East Greenland Current and the East Icelandic Current), and warm, more saline Atlantic Water from the south via the Irminger Current. This leads to a highly variable hydrographic environment, as the proportions of Arctic and Atlantic waters can fluctuate from year to year^10,11^. Up to eight distinct water masses can be characterized in this region in relation to changes in water temperature and salinity by surface heating, melting ice, mixing with warm Atlantic water and convection^10,12,13^. Over the last few decades, there has also been an increase in the salinity and temperature of the North Icelandic Irminger Current^10,14,15^. There is some evidence around Iceland that different diatom assemblages are linked with polar and Atlantic water masses based on sediment samples^16^ and that the southern coastal waters, primarily influenced by Atlantic waters, have higher overall primary productivity than the northern, polar-influenced, waters^17^. This suggests that further investigation into the distribution of phytoplankton taxa may reveal biogeographic patterns which could lead to a better understanding how long term increases in water temperature may impact marine primary producers around the island.

Until now, most phytoplankton diversity research in Icelandic waters has been based on microscopic identification. These studies have shown spring blooms to be dominated by diatoms, dinoflagellates and prymnesiophytes^18,19^. Due to the limitations of light microscopy, the known diversity of phytoplankton in this region is biased towards microphytoplankton (> 20 µm), and, to a lesser extent, nanophytoplankton (3–20 µm). As we are becoming more aware of the global importance of picophytoplankton in primary productivity^20–22^ and as indicators of warming waters^23^, it is important to use methods that can also detect these small size classes. Metabarcoding based on the amplification and sequencing of marker genes from DNA extracted from filtered seawater samples (also called metabarcoding) provides a high level of information and can be a powerful tool to resolve phytoplankton biogeography (e.g.^24^). This method has advantages over microscopy in revealing novel diversity of uncultivated microorganisms, of nano- and picophytoplankton and in resolving cryptic species^25–29^, which makes it a fitting method to further explore the biogeography of phytoplankton around Iceland.

The aim of this study is to get a broad overview of the diversity of eukaryotic phytoplankton around Iceland based on metabarcoding, and establish how it relates to the hydrographic environment over different seasons and years. To achieve this, we sequenced the hypervariable region V4 of the 18S ribosomal RNA gene and examined the relationship between community composition and water mass characteristics. Samples were taken for the full water column at 19 fixed stations around Iceland during seven annual spring surveys from 2012 to 2018, as well as one summer and one winter survey in 2017 and 2018, respectively.

## Material and methods

### Sample collection and environmental metadata

**Figure 1:**
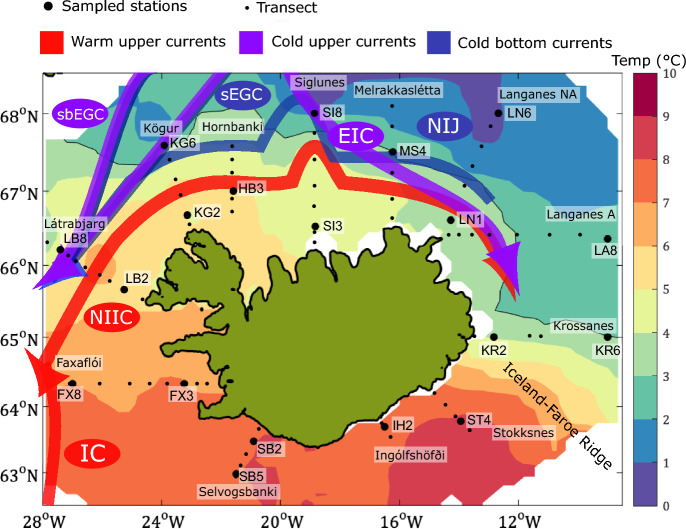
Maps of stations sampled around Iceland. Named section lines radiate outwards and stations are plotted at regular intervals, with numbers starting closest to the coast. The stations sampled in this study are plotted as larger black dots with labels. The average water temperature is plotted for the euphotic zone in all surveys (2012–2018). The map was created using MATLAB and GEBCO_2014 grid bathymetry data (https://www.gebco.net/data_and_products/gridded_bathymetry_data/version_20141103/) and SeaDataNet hydrographic data (https://cdi.seadatanet.org/search, search point Marine Research Institute). Temperature consistently increases with latitude, and the 6 $^{\circ }$C contour line divides north from south. The warm upper currents (red) constitute the Irminger Current and the North Icelandic Irminger Current, Cold upper currents (purple) constitute the East Greenland Current and Cold bottom currents consist of the North Icelandic Jet are all plotted as arrows indicating the distribution of waters around Iceland. These superimposed arrows were adapted with permission from “Alongstream, seasonal and interannual variability of the North Icelandic Irminger Current and East Icelandic Current around Iceland” by Casanova-Masjoan et al.^10^, Copyright (2020) American Geophysical Union.

A total of 929 samples were collected during annual oceanographic surveys conducted by the Marine and Freshwater Research Institute (MFRI) in May between 2012 and 2018 (spring), August 2017 (summer) and February 2018 (winter). These surveys sampled fixed oceanographic stations set on section lines that radiate outward from Iceland’s coast and across the shelf (Fig. 1). The lines are named after geographical features and abbreviated accordingly (e.g. Siglunes is SI), and the stations are numbered starting at the coast and increasing outward, which means that SI3 would be a relatively shallow station and SI8 would be situated in deeper waters (Fig. 1). For the purpose of this study, we selected one station relatively close to the coast and one station close to the shelf break for most lines, and 12–19 stations were sampled at each survey.

Water was sampled using a rosette equipped with a Sea-Bird 911+Plus Conductivity Temperature Depth (CTD) sensor with several 5-L Niskin bottles. A table of all the samples can be found at Figshare (10.6084/m9.figshare.21433011). The rosette was deployed at the fixed oceanographic and coastal stations surrounding Iceland from surface to bottom depths (Fig. 1). The conductivity sensor was calibrated using seawater samples that had their salinity measured on an Autosal 8400B salinometer. The CTD accuracies were 0.3 db, 0.001 $^{\circ }$C, and 0.002 for pressure, temperature, and practical salinity, respectively. For microbial analyses, one liter of seawater from the bottle sample was filtered onto 0.22 µm Sterivex filters and flash frozen in liquid nitrogen, then stored at − 80 $^{\circ }$C until further processing. Sample salinity and temperature measurements were taken for each sample and phosphate, nitrate and silicate concentrations were measured for most samples following the methods of Olafsson et al.^30^.

### Water masses within the euphotic zone

To identify the different water masses existing in the area we followed the criteria used in Casanova-Masjoan et al.^10^, which builds on the definitions reported in Rudels et al.^12^ and Våge et al.^13^ (Table 1). Since we were only studying the relationship of water masses with photosynthetic protists, we focused on water masses found within the range of an euphotic zone (0–110m).

**Table 1:** The main phytoplankton ASVs.

Division	Class	Genus	Species	ASV #	ASV (n)	ASV reads	ASV/Gen (%)	Gen/Class (%)	Class/Photos (%)
Ochrophyta	Bacillariophyta	*Asterionellopsis*	*Asterionellopsis glacialis*	asv00263	1	21,429	100.0	1.3	45.5
		*Chaetoceros*	*Chaetoceros socialis*	asv00023	94	94,259	48.2	11.5	45.5
		*Fragilariopsis*	*Fragilariopsis sublineata*	asv00476	33	34,203	88.9	2.3	45.5
			*Fragilariopsis cylindrus*	asv00044	33	3869	10.1	2.3	45.5
		*Proboscia*	*Proboscia alata*	asv00069	2	35,749	99.4	2.1	45.5
		*Pseudo-nitzschia*	*Pseudo-nitzschia sp.*	asv00061	19	49,291	47.6	6.1	45.5
			*Pseudo-nitzschia sp.*	asv00124	19	18,438	17.8	6.1	45.5
			*Pseudo-nitzschia multiseries*	asv00235	19	14,379	13.9	6.1	45.5
		*Thalassiosira*	*Thalassiosira rotula*	asv00004	49	441,903	73.1	35.5	45.5
	Bolidophyceae	*Triparma*	*Triparma pacifica*	asv00058	19	56,390	59.3	93.7	2.7
			*Triparma laevis clade*	asv00082	19	26,360	27.7	93.7	2.7
	Dictyochophyceae	*Apedinella*	*Apedinella radians*	asv00086	7	18,051	97.3	34.4	1.4
	Pelagophyceae	*Pelagomonas*	*Pelagomonas calceolata*	asv00210	1	12,105	100	17.9	1.8
Chlorophyta	Chloropicophyceae	*Chloroparvula*	*Chloroparvula pacifica*	asv00101	8	57,403	82.5	100.0	1.9
			*Chloroparvula pacifica*	asv00496	8	7827	11.2	100.0	1.9
	Mamiellophyceae	*Bathycoccus*	*Bathycoccus prasinos*	asv00009	12	247,623	99.0	38.8	17.2
		*Micromonas*	*Micromonas commoda A2*	asv00008	17	263,561	69.5	58.9	17.2
			*Micromonas polaris*	asv00033	17	62,603	16.5	58.9	17.2
			*Micromonas clade B3*	asv00051	17	44,877	11.8	58.9	17.2
Haptophyta	Prymnesiophyceae	*Emiliana*	*Emiliana huxleyi*	asv00068	3	51,660	97.7	5.7	24.9
		*Phaeocystis*	*Phaeocystis pouchetii*	asv00003	39	651,115	90.9	76.6	24.9
			*Phaeocystis antarctica*	asv00072	39	35,847	5.0	76.6	24.9
Cryptophyta	Cryptophyceae	*Plagioselmis*	*Plagioselmis prolonga*	asv00056	6	52,923	86.5	59.1	2.8

Taxonomic names were assigned via DADA2 using the PR2 database. ASV/Gen is the percentage contribution of the ASV reads to the total number of genus reads. Gen/Class is the percentage contribution of the Genus reads to the total number of class reads. Gen/Photo is the percentage of genus reads out of total photosynthetic reads for the dataset. Class/Photo is the percentage of class reads out of total photosynthetic reads. ASVs (n) is the number of ASVs found within that genus.

### DNA extraction and PCR amplification

DNA was extracted from the Sterivex filters using a modified protocol of the Epicentre Masterpure DNA Purification Kit (Madison, WI, USA) with two added mechanical lysis steps: freeze-thawing and bead beating, and a longer lysis incubation. Briefly, 600 µL of Tissue and Cell Lysis Solution (with 2 µL 50 mg/mL Proteinase K) were added directly into the Sterivex filter capsule, followed by an incubation period of 30 min at 65 $^{\circ }$C on a rotating mount. The lysate was frozen at − 80 $^{\circ }$C for 30 min and defrosted at 65 $^{\circ }$C for 10 min. The lysate was cooled on ice and 0.2 mg of 0.1 mm Silica beads (BioSpec, USA) added for 30 s of bead beating at 30 Hz. The samples were cooled on ice for 2 min before another 30 second bead beating step. The collected supernatant was transferred to clean Eppendorf tubes and the DNA extraction proceeded following the manufacturer’s instructions. DNA was resuspended in 50 µL 10 mM Tris (pH 8.00) and stored at − 80 $^{\circ }$C.

We amplified the hypervariable V4 region of the 18S rRNA gene (380 bp) using V4_18S_Next_F (5′-CCA GCA SCY GCG GTA ATT CC-3′) as forward and V4_18S_Next_R (5′-ACT TTC GTT CTT GAT YRA TGA-3′) as reverse primers, respectively^27,31^. The samples were amplified with 10 ng of template DNA using the protocol for Q5 high fidelity polymerase with GC enhancers (New England Biolabs) and an annealing temperature of 52 $^{\circ }$C. Polymerase chain reactions were done in triplicate and pooled. Illumina libraries were prepared using MagBio beads (London, UK) for PCR-cleanup and the Nextera XT Kit for index PCR (Illumina) following the MiSeq protocol for 16S rRNA sequencing (https://tinyurl.com/yp6r99zw). The samples were sequenced on the MiSeq Illumina platform (2 $\times$ 300 nt paired end) using the V3 Reagent Kit. All the samples were sequenced over the span of five different MiSeq sequencing runs (Illumina). The fastq files and associated sample depth, temperature and salinity data were deposited in the European Nucleotide Archive (ENA) at EMBL-EBI under accession number PRJEB44722 (https://www.ebi.ac.uk/ena/browser/view/PRJEB44722).

### Identifying amplicon sequence variants

Processing of raw reads, statistical analyses and generation of figures was done using R (V3.5.1; www.R-project.org). The dada2 package [V1.9.1]^32^ was used to quality trim reads and identify Amplicon Sequence Variants (ASVs). Reads were truncated at 230 nt (R1) and 215 nt (R2) after inspecting the read quality profiles of the lowest quality run, leaving a 20 nt overlap after pairing. Reads were filtered and trimmed using standard dada2 parameters with a maxEE value of 2 and a truncQ value of 2. The dada2 pipeline calculates error rates for each dataset using the first billion bases, after which sequences are dereplicated and ASVs are inferred. Paired reads were merged and sequences shorter than 350 bp and longer than 400 bp were removed, as the target amplicon length is around 380 bp. Chimeras were removed using a de novo approach, leaving about 90% of the reads, and ASVs represented by only one sequence (singletons) were removed. Taxa were assigned using a naíve Bayesian Classifier algorithm^33^) against the curated Protist Ribosomal Reference database [PR2, V4.12.0]^34^.

### Diversity and community composition analyses

Downstream analyses were performed using the *phyloseq* package for diversity and Principle Coordinate Analyses [V1.26.0]^35^, *ggplot2* for creating figures [ggplot2] and *treemapify* for treemaps.

To focus on genotypes that were more likely to be biologically relevant, we removed ASVs that only occurred in one sample. This left us with 99.5% (n = 41,118,276) of the original number of sequences and 31.6 % (n = 10,959) of the original number of ASVs. Metazoa and Fungi were excluded from the dataset to investigate only protist diversity, leaving 920 samples, 10,227 ASVs, and a median sequencing depth of 27,906 reads.

For the purpose of exploring patterns in phytoplankton diversity and relative abundance, we removed dinoflagellates from our dataset, as its high relative read abundance was obscuring the patterns of other taxa. Moreover, a large fraction of dinoflagellate species are heterotrophic, even within genera that are mostly autotrophic^36^ and dinoflagellate genomes contain large numbers of the rRNA operon^37^, which would leads to an overestimation of their real abundance compared to other plankton groups. We focused on five divisions within the euphotic zone which are primarily photosynthetic: Haptophyta, Ochrophyta, Cryptophyta, Chlorophyta and Rhodophyta. We removed the class Chrysophyceae as it was mainly represented in our dataset by known heterotrophic taxa (*Paraphysomonas* and uncultured taxa from clades H, I, C and F). The classes Bangiophyceae and Florideophyceae (Rhodophyta), and Xanthophyceae and Phaeophyceae (Ochrophyta) were also removed as they represent multicellular alga.

Only the samples from 0 to 110 m were studied to reduce the effect of limited light availability on community composition at lower depths (621 samples, 1395 ASVs, median sequencing depth = 6037 reads). In addition, prior to comparing the taxonomic composition of samples, the samples were normalized to the median sequencing depth. Due to the compositional nature of the sequence dataset, read abundance was used to infer abundance of phytoplankton in relation to each other (i.e. their relative abundance) for the purpose of identifying those that are dominant in the environment.

Since the most sampled season in this dataset was spring, we used these data to understand the geographic dimension in phytoplankton distribution without the influence of seasonal variation. For an overview of phytoplankton taxa in the Icelandic marine environment, taxa were assessed based on read abundance from all samples combined. For example, the top eight most dominant classes were calculated from total photosynthetic reads within the spring 2012–2018 dataset, which contributed to more than 1% of total photosynthetic reads.

To identify taxa that were dominant but also strongly influenced by their environment (i.e. restricted in distribution), we calculated the top four most dominant genera for each station and merged the results which culminated into a list of 12 dominant genera for the spring dataset (2012–2018). Only genus-assignments with a dada2 bootstrap value larger than 80 were investigated.

### Biogeography and environmental relationships

To investigate the relationship between taxa and environmental variables, the mean number of reads per sample (for all surveys 2012–2018) was plotted against geographical (i.e. station) and environmental parameters (water mass, temperature, salinity, phosphate, nitrate and silicate). For continuous variables the means for bins were calculated at selected intervals. To confirm the observed relationships, a Principal Component Analysis (PCA) was done using the packages *factoExtra*^38^, *factoMiner*^39^ and *missMDA* to impute missing values^40^.

To test if different water masses had distinct phytoplankton community compositions, the Aitchison distance was used to calculate beta diversity. This is an Euclidean distance calculated from centered log-ratio transformed data on ASV count data [*vegan* package]^41^ with the purpose of reducing the compositional bias already present in metabarcoding data^42,43^. A Principle Coordinate Analysis (PCoA, *phyloseq*) based on the Aitchison distance was done to investigate the impact of water masses and CTD in-situ temperature on phytoplankton community composition. The *DESeq2* Bioconductor package was used^44^ to determine which ASVs were differentially abundant between sample groups based on selected hydrographic parameters.

All scripts used for the data analyses and figure generation, as well as the ASV table, are stored at Figshare (10.6084/m9.figshare.21433011).

## Results

The V4 region of the 18S rRNA gene was sequenced for 929 samples collected around Iceland (Fig. 1) primarily in spring from 2012 to 2018, with two additional surveys in summer (August 2017) and winter (February 2018). Sequences from all depths were investigated for protist diversity, whereas only samples from the euphotic zone (0–110 m) were investigated for phytoplankton diversity. To study the relationship of phytoplankton composition in relation to water masses, water mass characteristics were assigned to samples collected from the euphotic zone.

### Oceanography in the euphotic zone

Five of the eight water masses observed by Casanova-Masjoan et al.^10^ were identified in our dataset within the 0–110 m euphotic zone. Surface Water occupies the upper meters of the overall area and has a wide range in temperature ($> 3 ^{\circ }$C). South of Iceland, the main water mass is the warm ($> 3 ^{\circ }$C) and saline (> 34.9) Atlantic Water, which is partially transformed by convection into colder Atlantic-origin Overflow Water (0 $^{\circ }\hbox {C}< \theta <3 ^{\circ }$C) on the Nordic Seas. Atlantic-origin Overflow water can be found on the East Icelandic Current and on the East Greenland Current (Fig. 1). On the northern side of Iceland, Polar Surface Water (< 0 $^{\circ }$C) and warm Polar Surface Water (0 < T < 3 $^{\circ }$C) are formed at high latitudes as melted ice, river run-off and surrounding oceanic waters mix. Average water temperatures over the 2012-2018 surveys show water temperatures decline with increasing latitude (Fig. 1). The temperature threshold between 5 and 6 $^{\circ }$C is roughly situated around the midline of the island, although warmer water extends to the northeast of Iceland due to Atlantic water travelling along the North Icelandic Irminger Current as it curves clockwise around Iceland (red arrow in Fig. 1).

### Diversity of marine protists within the water column

**Figure 2:**
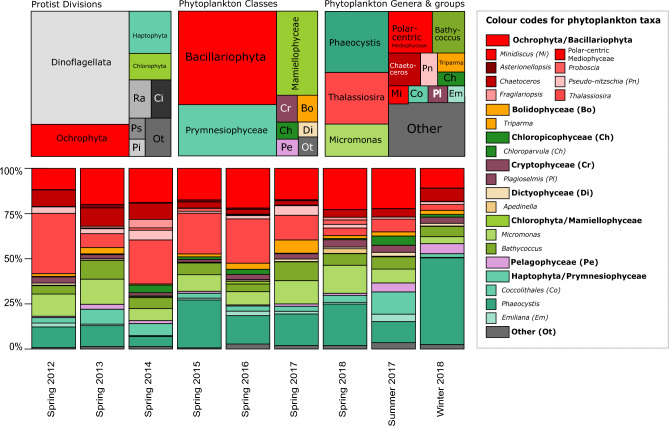
The relative read proportion of protist divisions. Left: full dataset, including all sampling depths. Note the disproportionally high representation of dinoflagellates. Ra, Radiolaria; Ci, Ciliophora; Ps, Pseudo-fungi; Pi, Picozoa. Middle and right: photosynthetic class and genus reads (only incorporates samples from euphotic zone, 0–110 m, and photosynthetic divisions). At the bottom, a bar plot showing the relative abundance of the most abundant phytoplankton classes and phytoplankton genera (sum of reads) over the surveys and seasons.

When all depths were considered, Dinoflagellata dominated protist read abundance (54.7%, Fig. 2) and were followed by photosynthetic divisions: Ochrophyta (15.4%), Haptophyta (8.7%) and Chlorophyta (5.5%, Fig. 2). Ciliophora and Radiolaria made up 3.7% and 4.1% of total protist reads, and pseudofungi and Picozoa represented 1.7% and 1.4% of total protist reads (Fig. 2).

To explore patterns in phytoplankton diversity and relative abundance, we focused on five divisions within the 0–110m zone which are primarily photosynthetic: Haptophyta, Ochrophyta, Cryptophyta, Chlorophyta and Rhodophyta (Fig. 2) and excluded Dinoflagellata plus other known heterotrophic taxa (see “Material and methods”). These photosynthetic divisions were dominated by the classes Bacillariophyceae, Prymnesiophyceae and Mamiellophyceae (45.7%, 25.1% and 17.3% of total reads, Fig. 2, Table 1), followed by Cryptophyceae, Bolidophyceae, Chloropicophyceae, Pelagophyceae and Dictyochophyceae (Fig. 2).

**Figure 3:**
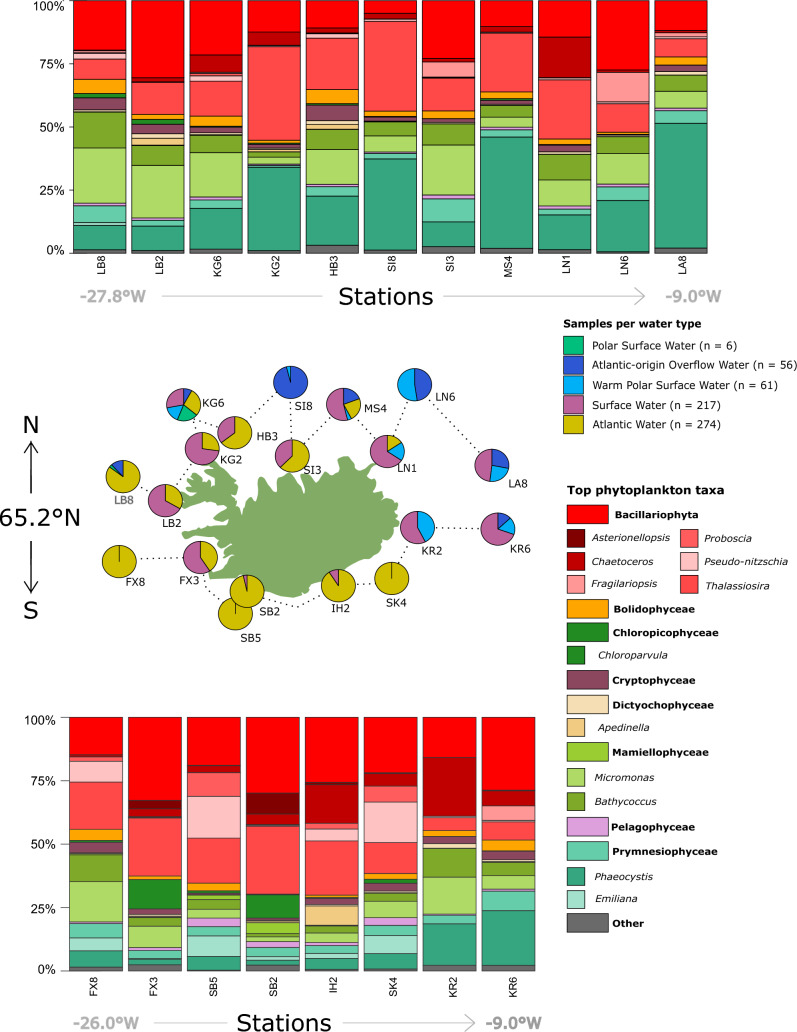
Geographical distribution of top phytoplankton classes and genera over the spring dataset (2012–2018) as well as the general trends in water mass distribution. Stations above 65.2$^{\circ }$N are plotted above the map of Iceland, and stations below 65.2$^{\circ }$N are plotted below the map of Iceland. The y-axis is ordered by longitude and follows the order indicated by the dashed line on the map. The bar plots show the relative abundance of reads for each station (total number of reads for spring 2012–2018). The pie charts around the map of Iceland indicate the proportion of samples assigned to different water masses within the 0–110 m water column based on their metadata. The water mass legend also indicates the total number of samples within the spring dataset assigned to those water masses.

**Figure 4:**
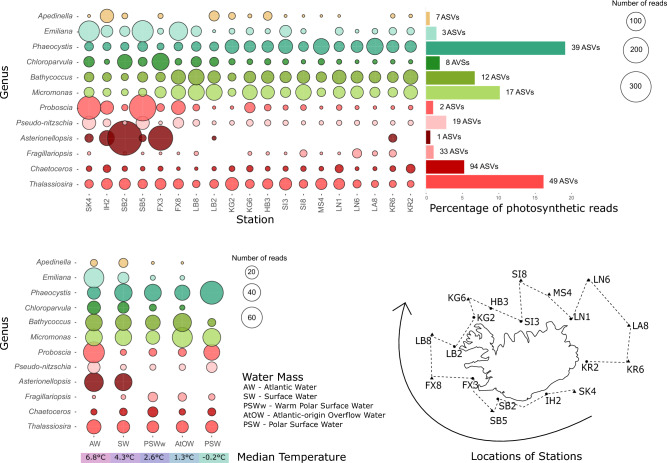
Average number of reads per sample (all surveys) for the 12 dominant genera plotted at each station (top) and water mass (bottom). The percentage of total photosynthetic reads and number of ASVs assigned per genus is indicated in the top bubble plot. The median water temperature for each water mass is indicated in the bottom bubble plot. A map of Iceland indicates the order of the stations, starting at the Iceland-Faroe Ridge. Most genera are ubiquitous, although some are absent from the north-eastern stations and polar-influenced water masses (e.g. *Asterionellopsis*).

To identify important phytoplankton taxa around Iceland, dominant genera were identified based on their relative read abundance in the complete spring dataset (Fig. 2 and Table 1). For the purpose of identifying dominant taxa with restricted distributions around the island, a merged list of the top four most dominant phytoplankton genera at each station (Figs. 3 and 4).

This merged list of dominant phytoplankton genera at each station contained 12 dominant genera, which was composed mainly of diatoms such as *Thalassiosira*, *Chaetoceros*, *Pseudo-nitzschia* and *Fragilariopsis* (Fig. 3, Table 1). The dictyochophyte *Apedinella* (Fig. 3, Table 1) was also found among the dominant taxa. Two prymnesiophytes were dominant in the dataset: *Phaeocystis* and the coccolithophore *Emiliania* (Fig. 3, Table 1). Three Chlorophyta genera, *Micromonas*, *Bathycoccus* and *Chloroparvula*, were also present (Fig. 3, Table 1). The first six genera in this list match the top six genera that were found to be overall abundant based on total reads (Supplementary Fig. S1). We also find genera that are overall abundant in Icelandic waters but do not dominate at particular stations: the diatoms, *Minidiscus* and *Actinocyclus* as well as *Chrysochromulina* (Prymnesiophyceae), *Triparma* (Bolidophyceae) and *Plagioselmis* (Dictyophyceae, Fig. S1). In contrast, *Apedinella* was in the top four most dominant genera at station IH2 but only 18th on the list for overall relative abundance (Figs. 3 and S1).

### Distribution and dynamics of dominant phytoplankton groups within the euphotic zone

#### Ochrophyta

Bacillariophyceae relative abundance appears lowest in winter in comparison to spring and summer (lower panel in Fig. 2), and higher south versus north of Iceland (lower panel vs upper panel in Fig. 3). *Thalassiosira* is by far the most dominant diatom genus in this class, followed by *Chaetoceros* (Fig. 2).

Most of the dominant diatom genera were present in all water masses and stations, except for the diatoms *Asterionellopsis* and *Fragilariopsis*. *Asterionellopsis* showed the most restricted distribution by only being present at seven of the 19 stations, mainly on the southwest coast of Iceland, and only in Atlantic and Surface Water (Figs. 3 and 4). Reads within this genus were all assigned to the species *A. glacialis*. *Asterionellopsis* had a higher average proportion of reads per sample for the temperature range 7–9 $^{\circ }$C, a pattern also noted for *Proboscia* (Supplementary Fig. S3). *Pseudo-nitzchia* also showed an increase in relative abundance around these temperatures, but for a slightly wider range of 7–11 $^{\circ }$C (Supplementary Fig. S3). These three diatom genera had higher average relative abundances at lower latitudes and in Atlantic Water, although *Proboscia* and *Pseudo-nitzschia* also had relatively higher relative abundances in Polar Surface Water (Fig. 4 and Supplementary Fig. S3). *Pseudo-nitzschia* and *Proboscia* seemed to increase in relative abundance with higher phosphate and nitrate values, whereas *Asterionellopsis* appears to be more abundant at lower nutrient values (Supplementary Fig. S3).

*Fragilariopsis* was present in all water masses except for a few stations at the south, west and east coast of Iceland (Figs. 3 and 4). *Fragilariopsis* was more dominant in colder water masses such as warm Polar Surface Water and Atlantic-origin Overflow Water as well as northern, oceanic stations such as LA6 and SI8 (Fig. 4).

One member of the Dictyochophyceae was represented in the 12 dominant phytoplankton groups, *Apedinella*, which only appear as one of the top four genera at one station, IH2 (Fig. 3). Bolidophyceae represented 2.7% of the photosynthetic reads and their dominant ASV, assigned to the genus *Triparma*, was the $8{\mathrm{th}}$ most abundant phytoplankton genus for the whole dataset (Supplementary Fig. S1). Bolidophyceae dominated at the northwestern stations LB8 and HB3, and in spring 2017 (Figs. 2 and 3).

#### Haptophyta

Two Haptophyta genera appear to play important roles in the phytoplankton dynamics around Iceland: *Phaeocystis* and *Emiliania* (Figs. 2 and 3). *Phaeocystis* represented the majority of Prymnesiophyceae reads (76.6%) is predominantly made up of a *P. pouchetii* ASV (90.9% of *Phaeocystis* reads. This was also the most abundant ASV out of all the photosynthetic divisions. The second most abundant *Phaeocystis* ASV was assigned as *P. antarctica*. The main *Emiliania* ASV represented 97.7% of the genus reads, likely representing the species *E. huxleyi* which is the most abundant coccolithophore in temperate and subpolar North Atlantic waters^45^.

The distribution of these dominant Prymnesiophyceae genera reflected the contrasting hydrographic environment around Iceland. *Emiliania* is more prevalent at southern stations influenced by relatively warm Atlantic and Surface Water, while *Phaeocystis* relative abundance increases to the northeast, peaking in Polar Surface Water (Fig. 4) which represents the coldest water mass (Supplementary Fig. S4). This inverted relationship is even more clear when plotted against temperature where the relative abundance of *Phaeocystis* starts to decrease at 5 $^{\circ }$C and *Emiliania* relative abundance per sample is at its highest around 5–9 $^{\circ }$C. *Emiliania’s* preference for warmer water is further reflected in its seasonal patterns as it is most dominant in summer and has the least relative abundance in winter (Fig. 2). *Phaeocystis*, on the other hand, dominates the phytoplankton community in winter (Fig. 2).

#### Chlorophyta

**Figure 5:**
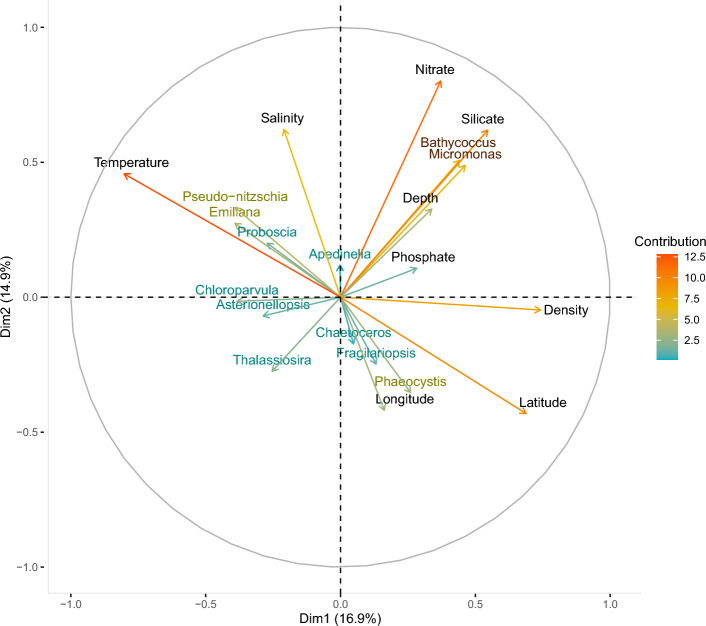
Principal Component Analysis depicting the relationship of the top 12 phytoplankton genera and various environmental parameters. Arrow length and colour indicates their level of contribution to variation. Temperature, salinity, water density, latitude, silicate and nitrate appear to be major environmental drivers of variation.

The most frequently observed Chlorophyta genera are *Micromonas* and *Bathycoccus*, both belonging to Mamiellophyceae, as well as *Chloroparvula* which belongs to the recently described class Chloropicophyceae^46^. *Micromonas* and *Bathycoccus* are also the third and fourth most abundant phytoplankton genera, after *Thalassiosira* and *Phaeocystis*, and together make up 84.4% of all Chlorophyta reads (50.9% and 33.6%, respectively) with *Chloroparvula* representing most of the rest (9.3%). *Micromonas* is dominated by an ASV that was assigned to *M. commoda* clade A2^28^. *Bathycoccus* has 99.0% of its reads assigned to *B. prasinos* by dada2. Both genera are widely distributed around Iceland in similar proportions with no particular biogeographic patterns (Figs. 3 and 4). One exception is that *Bathycoccus* has a conspicuously low proportion of reads in Polar Surface Water. Both *Bathycoccus* and *Micromonas* mean read counts per sample consistently increase as nitrate (NO$_{3}$), phospate (PO$_{4}$) and silicate (SiO$_{2}$) concentrations increase, which is reflected as a positive linear relationship in the PCA graph (Fig. 5 and Supplementary Fig. S3). These genera also show a strong positive relationship with silicate and depth (Fig. 5). It is likely that these patterns are better explained by low diatom abundance, reflected in the negative relationship on the PCA (Fig. 5), rather than a causal relationship with silicate concentrations.

*Choroparvula* shows an inverted relationship to *Micromonas* and *Bathycoccus* in the PCA as mean relative abundance decreases with increasing nutrient concentration (Fig. 5), and is also more abundant in southwestern stations and Atlantic and Surface Water masses (Fig. 4 and Supplementary Fig. S3). *Chloroparvula* mean read abundance clearly increases with temperature and it is most abundant in summer (Fig. 2 and Supplementary Fig. S3).

#### Cryptophyta

Cryptophyta represented only 2.8 % of photosynthetic reads (Table 1) with its dominant ASV assigned to *Plagioselmis* (Cryptophyceae) (Table 1). Cryptophyceae appear to have somewhat higher relative abundance in the northwest and west of the island (e.g. stations HB3, LB2 and LB8).

### Water mass and phytoplankton community composition for all seasons and years

Certain taxa are clearly different in relative abundance between the north and south of Iceland. Some were more dominant at Iceland’s southern coastal stations, mainly those that are influenced by Atlantic and Surface Water (Fig. 4). These taxa are *Apedinella*, *Emiliania*, *Chloroparvula*, and the diatoms *Proboscia*, *Pseudo-nitzschia* and *Asterionellopsis* (Fig. 4). These phytoplankton genera appear in the PCA on the negative side of the first axis, where increasing temperature is the main contributing variable (Fig. 5). Genera that show a more northern, or ’polar’ distribution are *Phaeocystis* and *Fragilariopsis* (Fig. 4). These genera also group together, along with *Chaetoceros*, on the positive axis of the first PCA axis where increasing latitude is one of the top contributing variables (Fig. 5).

**Figure 6:**
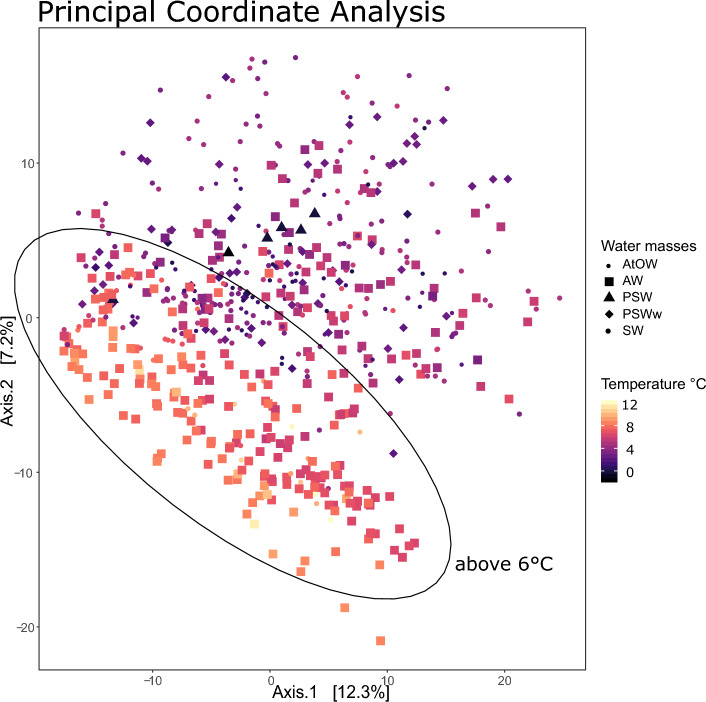
A Principal Coordinate Analysis of all phytoplankton samples (n = 621) based on the Aitchison distance between samples. The samples are coloured by temperature and the points are shaped according to water mass (Atlantic Water, Surface Water, Polar Surface Water, warm Polar Surface Water and Atlantic-origin Overflow Water). An statistical ellipse was added to indicate the clustering of samples at a temperature of 6 $^{\circ }$C and above. These samples mainly consist of Atlantic Water samples. Colder waters (below 6 $^{\circ }$C) consist of many different water masses with no clear grouping of samples.

The PCoA shows the impact of temperature and water mass on overall phytoplankton community composition and the most distinct cluster is formed for Atlantic Water at water temperatures above 6 $^{\circ }$C (Fig. 6). Clear clusters in community composition did not form for the other water masses and species composition mainly overlapped, although certain water types such as Polar Surface Water and Atlantic-origin Overflow Water had less variation in community composition (Fig. 6). This is likely because Polar Surface Water was assigned to the lowest number of samples of all the water masses (n = 6) due to being geographically restricted to only two stations and only being detected in spring 2013 and 2015, Supplementary Fig. S2). Atlantic-origin Overflow Water is mainly found in the oceanic north to east stations for the uppermost 0–110 m layer, representing a small geographic area, which could explain why phytoplankton community composition between these samples are fairly similar (Supplementary Fig. S2). Warm Polar Surface Water and Surface Water samples overlap almost completely in beta-diversity clustering (Fig. 6). These two water types are characterised by the same salinity thresholds but varying temperature, with a threshold at 3 $^{\circ }$C (Table 2. Warm Polar Surface Water and Surface Water overlap in geographic distributions from western to eastern stations (Supplementary Fig. S2).

**Table 2:** The threshold values used to assign water masses based on CTD temperature (T), salinity (S) and calculated water density ($\sigma$). Table is modified from Casanova-Masjoan et al.^10^.

Water mass	Acronymn	Threshold values
Surface water	SW	$\hbox {T} > 3\,\,^{\circ }\hbox {C}$
		$$\sigma _0 < 27.70\,\,\hbox {kg}/\hbox {m}^{3}$$
warm Polar surface water	PSWw	0 $\le \hbox {T} < 3\,\,^{\circ }\hbox {C}$
		$$\sigma _0<27.70\,\,\hbox {kg}/\hbox {m}^{3}$$
Polar surface water	PSW	$\hbox {T} < 0\,\,^{\circ }\hbox {C}$
		$$\sigma _0 < 27.70\,\,\hbox {kg}/\hbox {m}^{3}$$
Atlantic water	AW	T $\ge 3\,\,^{\circ }\hbox {C}$
		S > 34.9
Atlantic-origin overflow water	AtOW	0 $\le \hbox {T} < 3\,\,^{\circ }\hbox {C}$
		$$\sigma _0 \ge 27.80\,\,\hbox {kg}/\hbox {m}^{3}$$
		$$\sigma _{0.5} < 30.44\,\,\hbox {kg}/\hbox {m}^{3}$$

The threshold temperature values used for identifying water types are 0 $^{\circ }$C and 3 $^{\circ }$C (Table 2), but from the PCoA graph a threshold of 6 $^{\circ }$C better separates phytoplankton community composition in the Icelandic marine environment. This separation can be observed in both the physical and biological oceanography. When looking at the map of average water temperature for the 0–110 m water column across all surveys, the temperature threshold of 5 and 6 $^{\circ }$C appears to separate southern Atlantic-influenced waters and Arctic-influenced northern waters (Fig. 1). This is likely due to the median temperature of Atlantic Water being 6.8 $^{\circ }$C across the dataset, 2.5 $^{\circ }$C higher than the next warmest water mass, Surface Water (Fig. 4 and Supplementary Fig. S4). A differential abundance analysis further supports the biological separation between north and south, with waters colder than 6 $^{\circ }$C showing more relative abundance of polar species such as *Micromonas polaris*, *Chaetoceros neogracilis*, *Phaeocystis pouchetii* and *Porosira glacialis* and waters warmer than 6 $^{\circ }$C having a higher relative abundance in temperate phytoplankton species such as *Asterionellopsis glacialis*, *Cerautalina pelagica* and *E. huxleyi* (Table 3). The log2 fold change value (lfc) indicates how much higher or lower the read abundance of a particular ASV is, e.g., a value of 8.5 for *A. glacialis* means that this ASV has $2^{8.5}$ (362) times more reads for waters warmer than 6 $^{\circ }$C than for those colder than 6 $^{\circ }$C. Two major factors could be influencing this geographical separation of phytoplankton community composition: the different water sources (Atlantic vs. polar) where temperature values merely indicate the extent of the area the Atlantic water mass influences, or the direct impact of the actual *in situ* water temperature on the differential abundance of phytoplankton species.

**Table 3:**
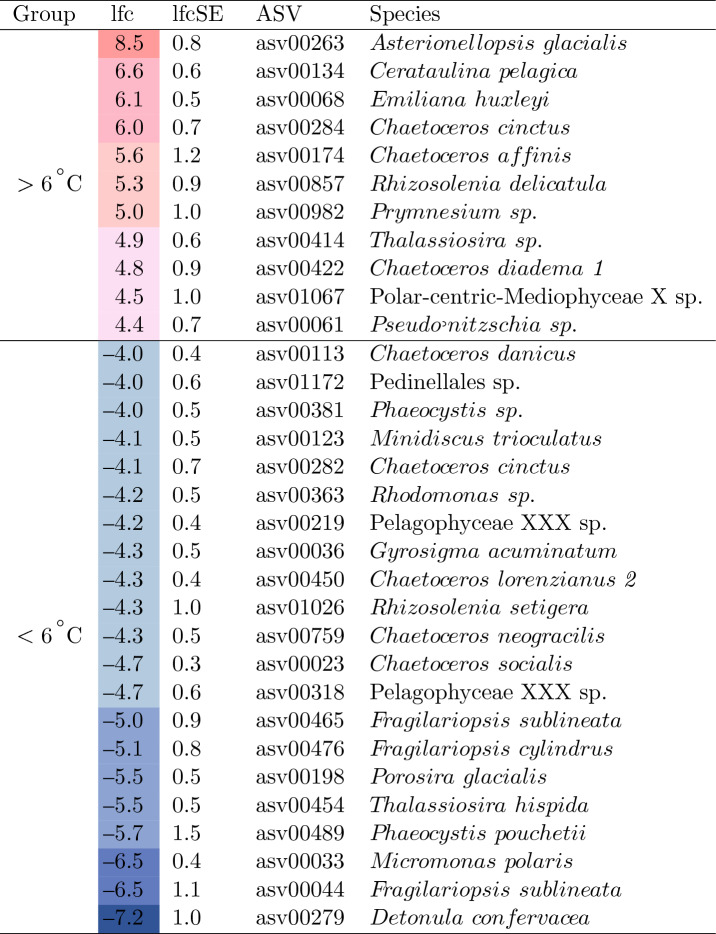
The ASVs which were found to be significantly different in their abundance between samples from water temperatures higher than 6 $^{\circ }$C and samples from temperatures lower than 6 $^{\circ }$C with log2foldchange (lfc) values higher than 4.

This value represents the magnitude to which read abundance differs between the two groups and lfcSE is its standard error. Taxonomic names were assigned via DADA2 using the PR2 database.

## Discussion

The species composition of marine microbes changes over short distances when contrasting water masses meet^8,9^. How the meeting of Atlantic and polar water around Iceland is reflected in phytoplankton biogeography and diversity has not yet fully been explored. Here we present an extensive overview of phytoplankton diversity around Iceland through the lens of metabarcoding and provide evidence that phytoplankton community composition is different between the northern and southern waters around the island.

### Southern distributed temperate taxa

Dominant genera that were more abundant in the warmer Atlantic and Surface Waters south and west of Iceland belonged to three phytoplankton divisions: Ochrophyta, Haptophyta and Chlorophyta. These were mainly diatoms (*Asterionellopsis*, *Proboscia* and *Pseudo-nitzschia*), as well as *Apedinella* (Dictyochophyceae), *Emiliania* (Prymnesiophyceae) and *Chloroparvula* (Chloropicophyceae). *Chloroparvula* and *Asterionellopsis* had very similar distributions, with their highest mean read abundance at southwestern coastal stations, SB2 and FX3. *Proboscia*, *Pseudo-nitzchia* and *Emiliania* also displayed similar distributions by being more abundant at the stations a little further from the coast in the southeast (SK4), south (SB5) and southwest (FX8) transects. *Apedinella* was one of the top four most abundant phytoplankton species at the south coastal station IH2, but was less prevalent at other stations and seemed to be absent from many stations that were further from the coast (SB5, FX8, LB8, LN6 and LA8). Jiang et al.^16^ studied the biogeography of diatom assemblages around Iceland using sediment samples and also found *Proboscia alata*, *Pseudo-nitzschia cf. turgitula* and *Pseudo-nitzschia seriata* to be more abundant in warmer waters in the south and west of Iceland.

*Asterionellopsis* represented the genus with the most restricted distribution, but it was also a genus consisting of a single species, *Asterionellopsis glacialis*. This species has been reported to grow in a wide range of temperatures and salinities^47,48^, so the reason for its restricted distribution around Iceland is not immediately clear. Both stations where it was most dominant were coastal and some of the more shallow stations in the study with bottom depths just under 100 m. *A. glacialis* is well adapted to dynamic areas, such as surf zones^49,50^, which could explain why it is more dominant in these coastal areas.

*Emiliania huxleyi* is known as a temperate bloom-forming coccolithophore, which is most abundant in summer in subpolar regions after the spring bloom had depleted most nutrients, such as silicate^51^. Our data indicates that *Emiliania* was more dominant in the warmer waters south from Iceland and in the summer survey. Multiple studies have shown that *E. huxleyi* is expanding polewards due to increasing water temperature and the influx of North Atlantic Waters, so that it has been defined as a tracer for Atlantic water in the Barents Sea^3,4^, e.g.

### Northern distributed polar taxa

A few genera indicated a preference for the northern, polar-influenced waters. In particular, *Fragillariopsis* and *Phaeocystis* had their highest mean relative abundances at temperatures ranging from − 1 to 5 $^{\circ }$C. *Fragilariopsis* taxa are polar diatoms, with species dominating the sea ice in both the Southern and Northern Hemisphere. *Fragilariopsis cylindrus* was identified as a good indicator for cold water^52^. Jiang et al.^16^ found *F. cylindrus* and *F. oceanica* to be more present in sediment samples collected north and northwest of Iceland and suggested that they could be potential indicators of melting ice and polar water. *Fragilariopsis* was at its lowest mean relative abundance at the highest salinity range of 34.8–35.2 in this dataset and the PCA also indicated a negative relationship with salinity (Fig. 5 and Supplementary Fig. S3), which could further support that it has a preference for the lower temperature and salinity associated with polar water.

*Phaeocystis pouchetii* and *P. globosa* bloom in the North Sea between early April and early June^53^, often having a competitive advantage over other phytoplankton groups after diatom blooms depleted the waters of silicate but have sufficient nitrogen left for their growth. *Phaeocystis pouchetii* abundance can increase with latitude in the north, where it has been observed in the northeast and Arctic waters^54^, 68–80 $^{\circ }$N. Degerlund and Eilertsen^54^ found *Phaeocystis* to co-occur with *Chaetoceros socialis* and *Fragilariopsis oceanica*, which is also reflected in our data through the clustering of *Phaeocystis* in our PCA with *Chaetoceros* and *Fragilariopsis* (Fig. 5). Our metabarcoding dataset indicates 39 genotypes within the genus, and while 90% of reads were assigned to *P. pouchetii* there was representation of other species too. Unexpectedly, *Phaeocystis antarctica*, which is a key *Phaeocystis* species in Antarctic waters, was the second most abundant *Phaeocystis* species in our dataset (Table 1).

### Pico- and nanophytoplankton

Whilst cell size can be very variable for phytoplankton species and was not measured in this study, taxonomic information can provide insight into the size ranges of well-studied groups. Many of the dominant genera in Icelandic waters fall within pico- and nanophytoplankton size ranges (0.2–2 µm, 2–20 µm), namely *Phaeocystis* (< 6 µm), *Emiliania* (< 5 µm), *Apedinella* (< 11 µm), certain *Fragilariopsis* species (< 10 µm, e.g. , Scott and Marchant, 2005), as well as the Chlorophyta genera: *Micromonas* (< 2 µm), *Bathycoccus* (< 2 µm) and *Chloroparvula* (< 3 µm). Our sequencing results show that the green algae, Chlorophyta, are important contributors to phytoplankton in Icelandic waters as the $3^{\mathrm{rd}}$ most dominant phytoplankton division. In most surveys, *Micromonas* was more abundant than some of the dominant diatom genera, such as *Chaetoceros*. This finding is in line with other molecular studies that have revealed green phytoplankton as key, or even, dominant, contributors in marine environments^29,55^. The presence of Chlorophyta has previously been detected in Icelandic marine waters as part of the Ocean Sampling Day dataset^55,56^, but other than these three coastal samples no study to date has investigated the biogeography of green picoalgae in the Icelandic marine environment. Mamiellophyceae (dominated by *Bathycoccus* and *Micromonas*) are usually the main green algae in coastal waters, whereas Chloropicophyceae tend to be more prevalent in nutrient-depleted (oligotrophic), oceanic waters^57^. Because of their small size and higher surface to volume ratios, picophytoplankton often have an advantage in oligotrophic waters as they are able to more easily obtain nutrients compared to larger species.

However, even within Chlorophyta group of picophytoplankton there appear to be contrasting distributions for *Chloroparvula* in comparison to *Micromonas* and *Bathycoccus* according to the PCA results and read abundance plots. While *Micromonas* and *Bathycoccus* were consistently abundant at each station, their relative abundance decreased at higher temperatures of 7–13 $^{\circ }$C, where *Chloroparvula* relative abundance increased. *Chloroparvula* seemed to be more dominant in samples with lower nutrient concentrations and higher salinity which ties into the findings of other studies in which Chloropicophyeae is more abundant in more saline and oligotrophic environments^46,55^.

As the majority of our analyses were based on samples taken during spring surveys, it is likely that phytoplankton bloom events could have resulted in high consumption rates of certain nutrients during this time. DNA also persists in the environment after cells have died which makes it difficult to establish the causal relationship between phytoplankton relative abundance and nutrient concentrations without sampling over a series of time. Silicate consumption can be used as an indicator of diatom population growth^58^ which could explain why there is a negative correlation between some of the diatom species (*Thalassiosira*, in particular) and silicate concentration (Fig. 5). The positive correlation between *Micromonas*, *Bathycoccus*, silicate concentration and depth suggests an advantage of picophytoplankton in waters where diatom abundance is low.

Bolidophyceae fall within the pico- and nano size range and had been detected in Icelandic waters before through the metabarcoding dataset of Ocean Sampling Day, which showed the contribution of Bolidophyceae to increase towards northern latitudes and *Triparma pacifica* and *Triparma eleuthera* were particularly abundant in polar waters^59^. Some species of *Triparma* have silica plates and along with coccolithophores and nanoplanktonic diatoms, such as *Minidiscus*, could play a much larger role in carbon export and biomineralization than previously estimated for nanophytoplankton^60,61^.

### Conclusion

This study shows that phytoplankton composition is different between the Atlantic- and polar-influenced waters to the south and north of Iceland, most clearly represented by a threshold temperature of 6 $^{\circ }$C. These two regions appear to represent polar and temperate environments for phytoplankton as certain representatives of these environments are differentially abundant in each. Some of these species can be potential indicators or tracers for the expansion or retraction of different water sources around Iceland, such as *E. huxleyi* for Atlantic water and *M. polaris* for polar water. This study presents the opportunity for further investigation into the biogeography of specific taxa in Iceland’s complex hydrographic environment.

## Supplementary Information

Supplementary Figures.

## Data Availability

The 18S rRNA fastq files and associated sample depth, temperature and salinity data were deposited in the European Nucleotide Archive (ENA) at EMBL-EBI under accession number PRJEB44722 (https://www.ebi.ac.uk/ena/browser/view/PRJEB44722).

## Code availability

RMarkdown documents containting the scripts for DADA2 processing and phyloseq figure generation were stored at Figshare (10.6084/m9.figshare.21433011).
