# Supplementary material for: The distribution and diversity of eukaryotic phytoplankton in the Icelandic marine environment
Source: Sci Rep. 2023 May 25;13:8519. doi: 10.1038/s41598-023-35537-2 (PMC10212971; doi:10.1038/s41598-023-35537-2)
Supplement: Supplementary file 1 — Supplementary Figures. [file 41598_2023_35537_MOESM1_ESM.pdf]

The distribution and diversity of eukaryotic phytoplankton in the Icelandic marine environment

Supplementary Material

Mia Cerfonteyn [1,2,3*], René Groben [1], Daniel Vaulot [4], Kristinn Guðmundsson [3], Pauline Vannier [1], M. Dolores Pérez-Hernández [5], Viggó Þór Marteinsson [1,2]

[1] Matís, Vinlandsleið 12, 113 Reykjavik, Iceland

[2] Faculty of Food Science and Nutrition, University of Iceland, Læknagarður, Vatnsmyrarvegur 16, 101 Reykjavik, Iceland

[3] Marine and Freshwater Research Institute, Fornubúðir 5, 220 Hafnarfjörður, Iceland

[4] Sorbonne Université, CNRS, UMR7144, Station Biologique de Roscoff, 29680 Roscoff, France

[5] Unidad Océano y Clima, Instituto de Oceanografía y Cambio Global, IOCAG, Universidad de Las Palmas de Gran Canaria, ULPGC, Unidad Asociada ULPGC-CSIC, Las Palmas de Gran Canaria, Spain

* Corresponding author: mecerf@gmail.com

Submitted to **Scientific Reports**

Date: April 23, 2023

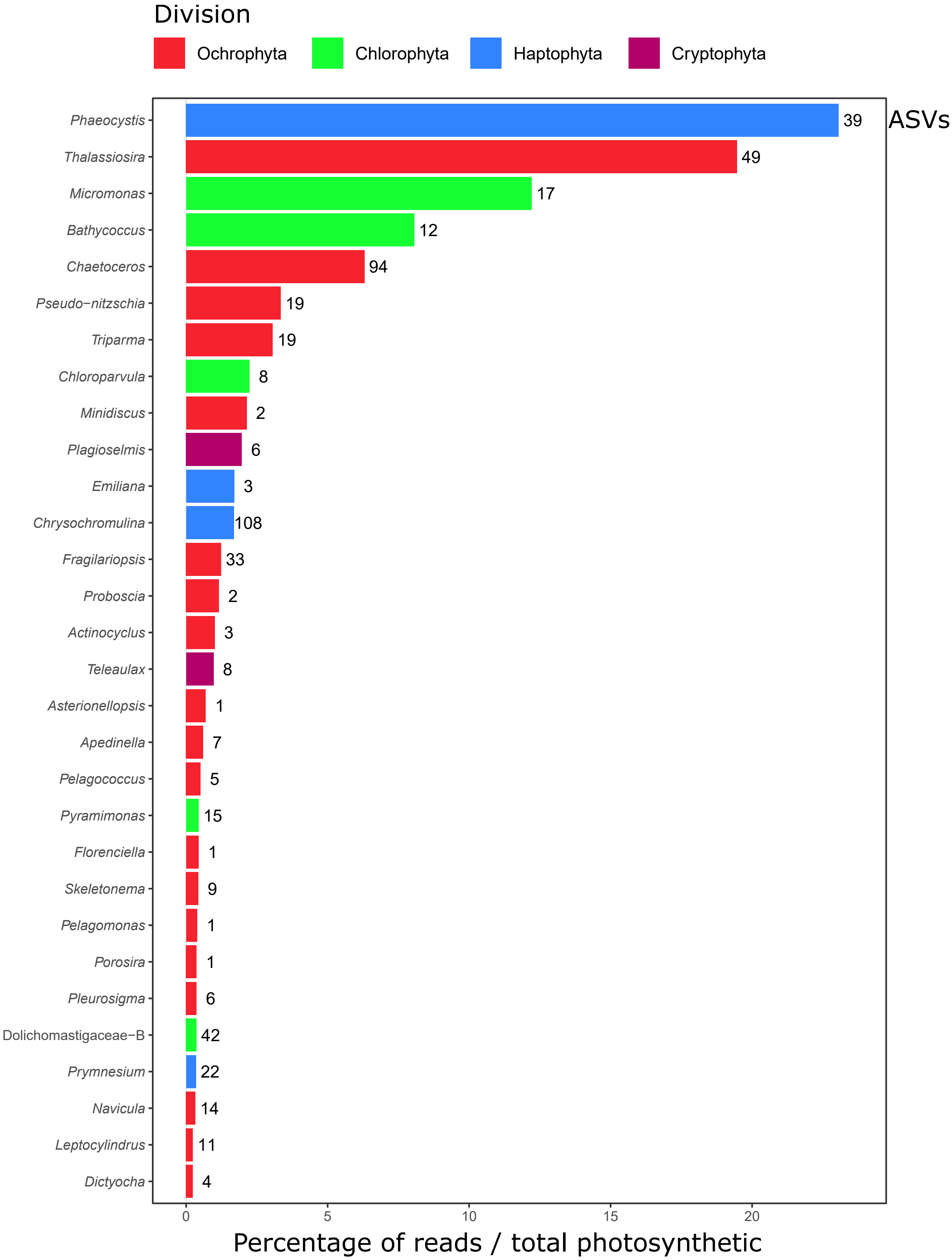

Figure S1: The percentage contribution to photosynthetic reads of the 30 most dominant phytoplankton genera within the dataset, as well as the number of ASVs found within each genus (right of each bar).

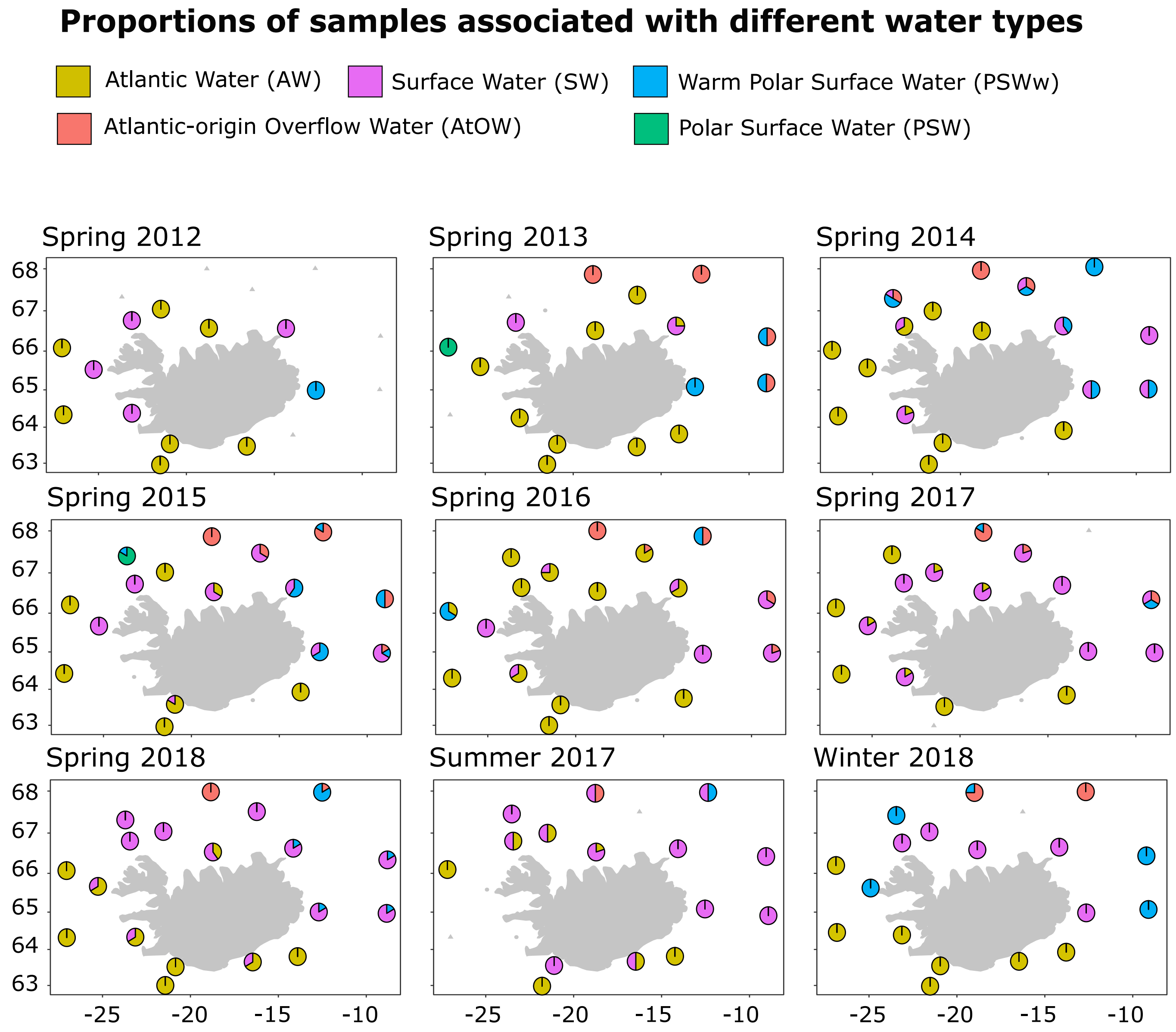

Figure S2: Samples were collected in different water masses found throughout the water column (0 to 110 m). The proportions (number of samples associated with each water mass) is plotted per station for each survey. The southern waters are consistently dominated by Atlantic and Surface Water, whereas the northern waters fluctuate more in their proportions of water types over the years for the same season (Spring). Fresher Surface Waters extend more to the south in Summer, whereas Winter has a higher proportion of warm Polar Surface Water and Atlantic-origin Overflow Water.

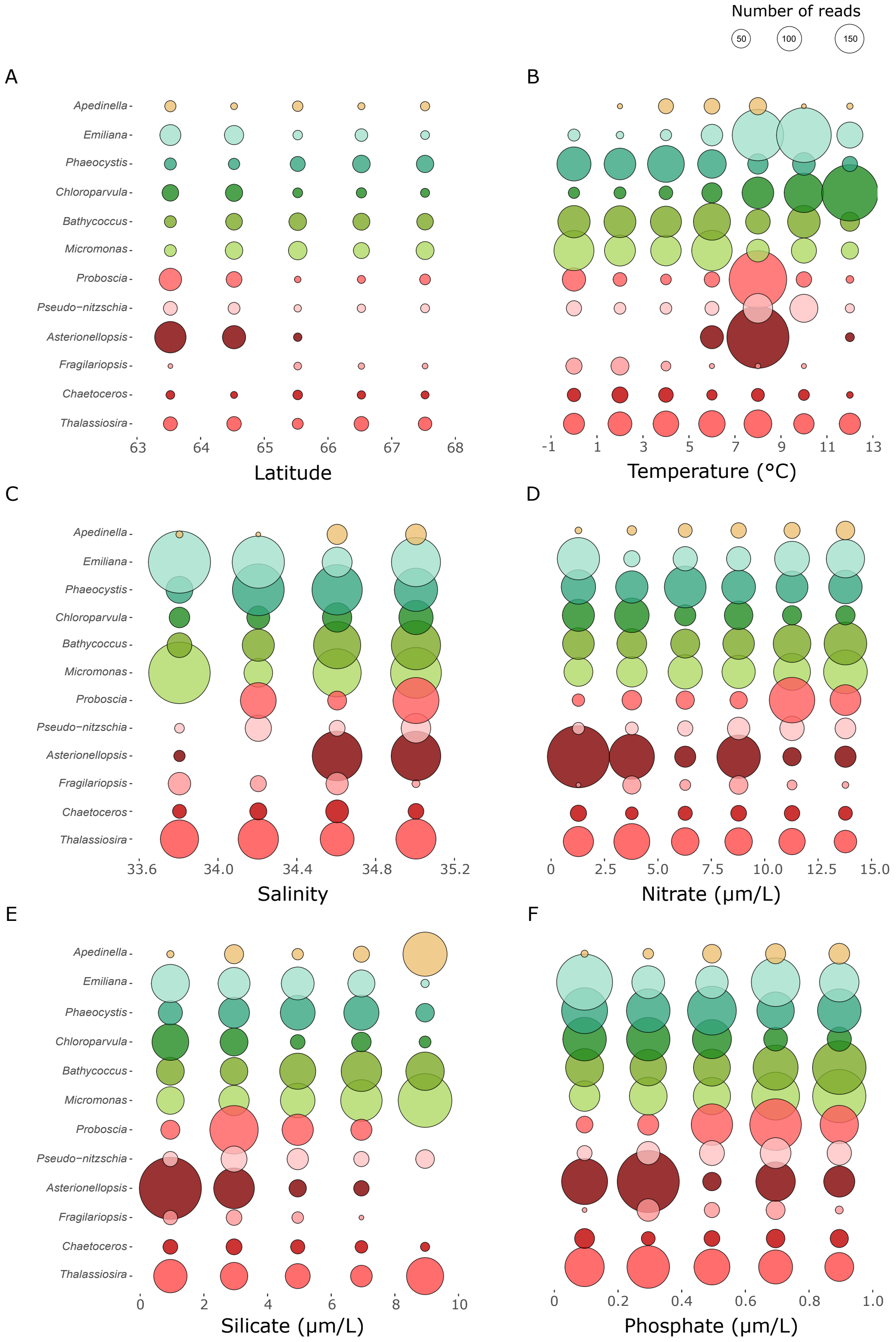

Figure S3: The average number of reads per sample for the top 12 phytoplankton genera plotted against different environmental factors.

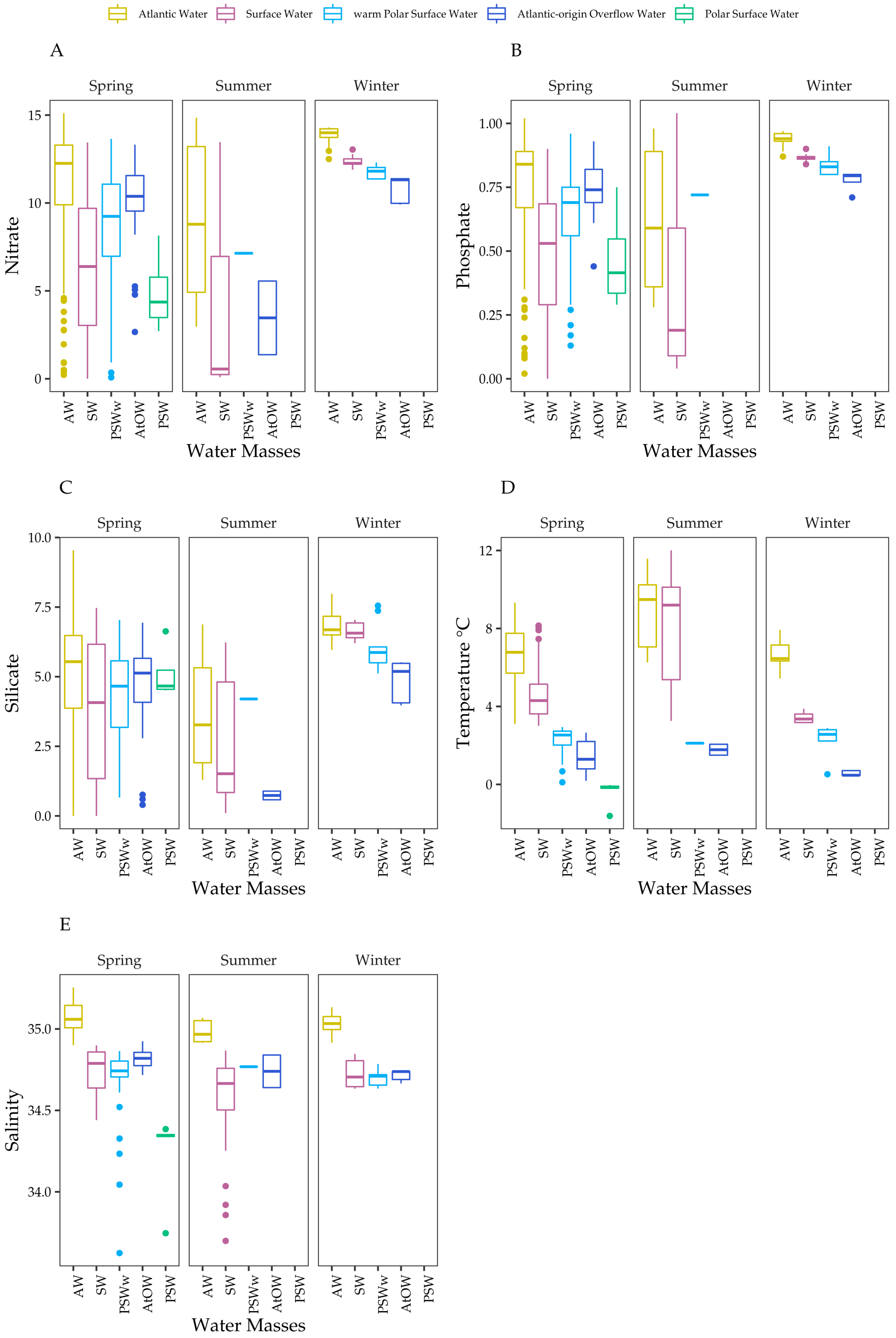

Figure S4: Average nutrients (phosphate, nitrate and silicate), temperature and salinity values for each of the water masses in the different seasons (spring, summer and winter). Atlantic water generally has the highest median nutrient, temperature and salinity values in all seasons. Winter nutrient values show less variation. Atlantic and Surface water temperatures are similar in spring and summer, but nutrient and salinity values are consistently different.

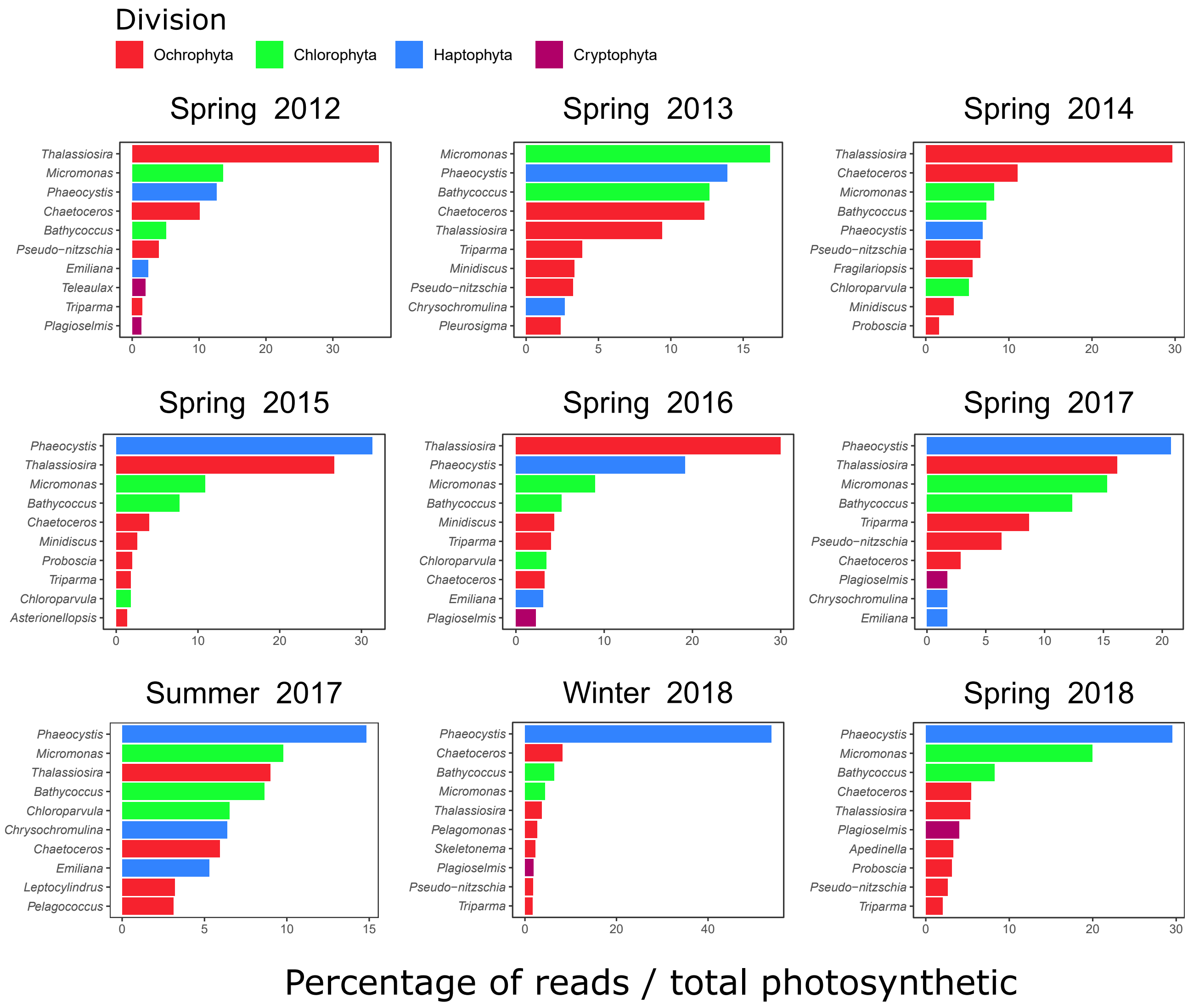

Figure S5: Top 10 contributing photosynthetic genera per survey. Percentages are calculated as number of genus reads / total photosynthetic reads for that survey.
